# Pupil Response to Affective Stimuli: a Biomarker of Early Conduct Problems in Young Children

**DOI:** 10.1007/s10802-020-00620-z

**Published:** 2020-01-25

**Authors:** Daniel T. Burley, Stephanie H.M. van Goozen

**Affiliations:** School of Psychology, Cardiff University, Cardiff, CF10 3AT UK

**Keywords:** Conduct problems, Pupillometry, Biomarker, Autonomic arousal, Emotion dysregulation

## Abstract

Childhood conduct problems have been associated with reduced autonomic arousal to negative cues indicative of an insensitivity to aversive stimuli, with mixed evidence in response to positive cues. Autonomic arousal to affective stimuli has traditionally been measured through galvanic skin responses and heart-rate, despite evidence that pupillometry is more reliable and practically beneficial (i.e., no wires are attached to the participant). The current study is the first to examine abnormal pupillary responsivity to affective stimuli as a biomarker for childhood conduct problems. We measured pupil reactivity to negative, positive and neutral images in 131 children aged 4–7 years, who were referred by their teachers for being at risk of future psychopathology. We assessed relationships between pupil response to the images and teacher-rated scores on the Strengths and Difficulties Questionnaire (SDQ), which indexed child conduct problems and their overlapping emotional, behavioural and social difficulties. Reduced pupil dilation to negative images was associated with significantly increased conduct, hyperactivity, emotional and peer problems, as well as reduced prosocial behaviour. Composite scores for disruptive behaviour and emotional difficulties both uniquely predicted blunted pupil response to negative threat stimuli; there were no relations with pupil responses to positive images. These findings highlight that blunted pupil responsivity to negative stimuli serves as a biomarker for early disruptive behavioural problems and affective difficulties. Pupillometry offers an inexpensive, fast and non-intrusive measure to help identify children who are showing early disruptive behaviour or experiencing affective difficulties, which can provide opportunities for preventative intervention to avoid further psychopathology.

**Electronic supplementary material:**

The online version of this article (10.1007/s10802-020-00620-z) contains supplementary material, which is available to authorized users.

Physiological changes in response to emotional stimuli are fundamental to the emotional experience and adaptive regulation of physiological arousal is vital for healthy functioning (Scarpa 2015). Increases in physiological arousal occur as a result of the activation of neural circuits responsive to potentially life-threatening aversive and life-sustaining appetitive stimuli that trigger a cascade of physiological (and cognitive) changes that guide psychological (i.e., affective, cognitive and interpersonal) mechanisms involved in behavioural tendencies to approach or avoid (Lang and Bradley 2010). The Research Domain Criteria (RDoC) framework identified ‘Arousal/Regulatory Systems’ as one of five higher-order domains of human functioning and behaviour, indicating that arousal in response to aversive and appetitive stimuli reflects the sensitivity of an organism to these cues (see https://www.nimh.nih.gov/research-priorities/rdoc/index.shtml).

Abnormal arousal responses and dysregulation may disrupt psychological mechanisms involving temperament and personality and place the individual at risk of maladjustment (Scarpa 2015). Indeed, research has linked abnormal physiological arousal (hypo- or hyper-arousal) in response to emotional stimuli to the onset and severity of a range of serious psychopathology (Hajcak and Patrick 2015), which includes specific clinical symptoms that cut across diagnostic boundaries (Yancey et al. 2015). This illustrates the potential of psychophysiological responsivity to account for comorbidity of clinical symptoms observed across (and heterogeneity observed within) certain traditional disorders (Baskin-Sommers and Foti 2015; Hajcak and Patrick 2015). Similar abnormal physiological processes exist early in development, as abnormal arousal in childhood has been linked to cognitive, emotional and behavioural difficulties (Scarpa 2015). This highlights the value of investigating the dimension of arousal within children as a biomarker of early maladjustment. It is important to identify difficulties early in development in order to disrupt pathways to later serious psychopathology.

## The Autonomic Nervous System and Pupillometry

The activity of the autonomic nervous system (ANS) is closely tied to emotions and the stress response, and abnormal autonomic activity has been linked to a range of neurodevelopmental difficulties (Hajcak and Patrick 2015). The ANS is comprised of the excitatory sympathetic nervous system (SNS) – responsible for the ‘fight or flight’ response and characterised by physiological reactions such as increased sweating, respiration and heart-rate - and the inhibitory parasympathetic nervous system (PNS), which is responsible for restorative physiological responses during periods of safety. Autonomic reactivity occurs when an aversive or appetitive motivational cue is presented, which triggers the sympathetic chain through activation of the basolateral and then central nucleus of the amygdala via projections to the lateral hypothalamus (Lang and Bradley 2010).

The excitatory SNS has typically been indexed through skin conductance reactivity (SCR) and heart-rate (HR) – although heart-rate is understood to be influenced by both SNS and PNS activation (Norman et al. 2014) - yet there are a number of advantages for measuring SNS activity using changes in pupil diameter. Firstly, measuring emotional arousal using the pupil does not require that wires are attached to the participant, which is particularly beneficial when working with apprehensive populations such as young children (and leads to a shorter procedure). Emotionally-modulated changes in pupil diameter occur rapidly (after approximately 500 ms) and so pupillometry offers a time-sensitive measure of autonomic arousal. In comparison, SCRs occur 1000–5000 ms after stimuli onset offering a more delayed index of sympathetic autonomic reactivity. Bradley et al. (2017) reported that the pupil is a more reliable indicator of emotional arousal compared to SCR. While both SCR and pupil responsivity indexed sympathetic autonomic arousal, 92% of participants evidenced increased pupil diameter for affective compared to neutral images, whereas only 67% of the participants showed this pattern according to SCR. Lastly, numerous individual differences factors (e.g. oily skin type, dampness, participant movement) affect the ability to obtain effective data using SCR and the number of ‘non-responders’ within the sample population (Venables and Mitchell 1996). Therefore, pupillometry offers a more temporally sensitive, reliable, and practical index of SNS activity.

Previous research has shown the value of pupillometry for measuring autonomic arousal as the pupil dilates in response to emotionally-laden stimuli compared to neutral stimuli and this has been demonstrated in response to images, sound-clips, facial expressions and video-clips (Bradley et al. 2008; Burley et al. 2017). Further, studies have demonstrated that abnormal pupil responsivity to affective stimuli across children and adults may act as a potential autonomic biomarker of neurodevelopmental difficulties, such as depression (Burkhouse et al. 2014; Burkhouse et al. 2015; Silk et al. 2007), anxiety (Burkhouse et al. 2014), bipolar disorder (Lemaire et al. 2014) and autism spectrum disorders (Nuske et al. 2014).

## Autonomic Arousal and Conduct Problems

Over the last few decades, a wealth of research has shown the relationship between reduced autonomic activity and different indexes of antisocial behaviour. Antisocial behaviour is a heterogeneous construct that encapsulates a wide range of co-occurring disruptive behavioural problems, including oppositional behaviour, conduct problems and aggression, and antisocial psychopathologies, such as psychopathy. Anti-sociality in early childhood is observed in the form of temper outbursts, hard-to-manage and rule-breaking behaviour, and aggression. The association between the spectrum of conduct problems and low ANS activity in response to negative emotional stimuli is well-established in adults and in children (Blair 1999; Fanti et al. 2019; Raine et al. 2000; Van Goozen et al. 2007). Moreover, longitudinal studies show that autonomic hypo-responsivity in infancy or early childhood is a biomarker for later aggression and antisocial behaviour (Baker et al. 2013; Gao et al. 2009, 2010). Low autonomic responsivity to negative cues is considered to reflect fearlessness triggered by an insensitive defensive motivational system (Blair 2006; Patrick and Bernat 2009). Indeed, Fanti et al. (2019) concluded in a meta-analysis that examining the activity of the SNS should be considered as a research priority when examining psychophysiological measures, as low SNS reactivity to threatening stimuli is an important indicator of childhood conduct problems. Research on autonomic responding to positive cues in children with conduct problems has not yielded a clear picture, with some studies finding reduced cardiovascular or electrodermal responsivity to reward in those with childhood conduct problems (Beauchaine et al. 2008; Crowell et al. 2006; de Wied et al. 2012; Gatzke-Kopp et al. 2015), whereas others found reduced responsivity regardless of valence (Herpertz et al. 2005).

The majority of studies to-date have explored SCR to affective stimuli and adopted group-based study designs, comparing children with a variety of conduct problems to control participants without conduct problems. This is problematic as antisocial behaviour is a heterogeneous construct that can be conceptualised along varying dimensions of behavioural and emotional processes (as described within the RDoC framework) and, therefore, treating all antisocial individuals (and, indeed, individuals in the control group) as a homogenous group masks important information across specific dimensions. This is particularly important given that conduct problems vary in severity and often co-occur with other externalising disorders, such as Attention Deficit Hyperactivity Disorder (ADHD), as well as wider neurodevelopmental difficulties such as callous features or internalising symptoms including anxiety (Beauchaine et al. 2010; Blair et al. 2013; Fanti et al. 2019; Lahey et al. 2008; Patalay et al. 2015) that can show independent and differing relationships to patterns of autonomic responsivity. It is therefore important to explore the dimensional relationship between childhood conduct problems, with their various behavioural (peer problems, impulsivity, poor behavioural control) and emotional (fearlessness, irritability, emotion regulation problems) components, and sympathetic autonomic reactivity in a large heterogeneous sample, in order to allow for a greater understanding about the specific influence of conduct problem as a dimensional neurodevelopmental construct (Fonagy and Luyten 2017).

Further, despite the association between conduct difficulties and low autonomic arousal, only one study has explored the relationship between pupil responses to affective stimuli in the context of antisociality and this was limited to an adult sample. Burley et al. (2018) found that adults high in psychopathy, specifically those with interpersonal-affective features (e.g. callousness, lack of remorse) showed reduced pupil diameter in response to negative threat stimuli, but normal pupil responses to positive stimuli. In contrast, lifestyle-antisocial features of psychopathy (e.g. impulsivity, poor behavioural control) were unrelated to pupil response to affective stimuli. Therefore, there is evidence that pupil hypo-responsivity to negative threat cues is a biomarker of certain antisocial features in adulthood that are linked to persistent and severe antisocial outcomes (McCuish et al. 2015). As yet, no study has investigated pupil responses to affective stimuli in young children showing early conduct problems, as well as wider disruptive behavioural problems. It is important that we examine this biomarker early in development as it may help to identify children early who are at risk of more severe conduct problems later.

## Current Study

The current study aimed to examine the role of abnormal autonomic arousal to affective stimuli in a sample of young children with emerging conduct and emotional problems. A further aim was to explore whether impaired responding was evident across both negative threat and positive appetitive stimuli reflecting the functioning of underlying defensive and appetitive motivation systems. We used a non-intrusive pupillometry paradigm to measure autonomic response to affective images in a large sample of young children (aged 4–7 years old) with emerging behavioural problems who are at risk of future psychopathology. In line with findings in adults with ASPD (Burley et al. 2018), we predicted a reduced pupil dilation to negative images in children with conduct problems and no association between conduct problems and abnormal pupil dilation to positive images. We also examined the specificity of these relations by investigating to what extent autonomic impairment was also observed in those with emotional, behavioural and/or interpersonal difficulties.

## Method

### Participants

One-hundred and thirty-one children (38 girls, 93 boys) aged 4–7 years old (*M* = 73.20 months, *SD* = 12.36, range = 49–95 months) were referred to the Neurodevelopment Assessment Unit (NDAU; http://www.cardiff.ac.uk/research/explore/research-units/neurodevelopment-assessment-unit) at Cardiff University by their schools for a range of socio-emotional, behavioural and cognitive difficulties. None of the children had received a diagnosis for any neurodevelopmental disorder at the time of testing (and therefore were not receiving any medication), although many were on the diagnostic pathway. Written informed consent was obtained from a parent or caregiver for each child. Each child and their parent/care-giver attended two assessment sessions at the NDAU, where the child completed a range of tasks, including the pupillometry task. All experimental procedures were given ethical approved by Cardiff University.

### Pupillometry Task

The children viewed affective and neutral images and their pupil diameter was measured in response. The images were selected from the International Affective Picture System (IAPS; Lang et al. 2008): Ten negative (mean valence/arousal based on IAPS normative ratings = 3.92, 6.05), ten positive (mean valence/arousal = 7.55, 5.89) and ten neutral images (mean valence/arousal = 5.28, 3.12) were presented. The negative and positive images were matched for arousal (*p* = .44), and were rated as more arousing than the neutral images (*p*s < .001). The images were converted to grey-scale and matched for luminance and luminance-contrast (*p*s > .05). Each image was presented for two seconds, before a brief tone indicated that the child could press a button to remove the image from the screen. Each image was preceded by a grey screen with a fixation cross in the centre for one second, and followed by a blank grey screen for three seconds; both of these slides were luminance-matched to the image set.

### Strengths and Difficulties Questionnaire

As part of the school referral process to the NDAU, the child’s teacher completed the Strengths and Difficulties Questionnaire (SDQ; Goodman 1997) prior to the child’s assessment session. The SDQ is a 25-item screening tool to assess the child’s functioning across emotional, conduct, hyperactivity/inattention and peer relationships problems, as well as examining prosocial behaviours. Missing SDQ item-scores were calculated based on the mean scores for the remaining items and rounded to the nearest whole number. Each subscale demonstrated decent internal reliability (Cronbach’s *α*: Emotional problems = .75, Conduct problems = .70, Hyperactivity = .73, Peer problems = .70, Prosocial = .83). The children within the sample showed a wide range of difficulties and 65.50% were rated by their teachers as having ‘High’ or ‘Very High’ difficulties according to their total SDQ scores (see for more information below on the SDQ).

### Lucid Ability Assessment

To explore the role of child intelligence on pupillary responses, an estimate of intelligence was obtained through the administration of the Lucid Ability assessment (Version 5.15; GL Assessment 2014). This task measures general conceptual ability across verbal and non-verbal reasoning tasks. For children younger than 7 years old, verbal ability was assessed by a picture vocabulary task and non-verbal ability was assessed via a mental rotation task. In children aged 7 years or older, verbal ability was assessed through a conceptual similarities task and non-verbal ability via a matrix problem-solving task. None of these tests requires the child to read as the instructions and tasks are read aloud to them. The child receives a standardised score based on their performance on the verbal and non-verbal tasks, as well as a score for general conceptual ability that is the combination of each task’s score. Four children did not complete the non-verbal reasoning tasks and their verbal reasoning task score was used as their general conceptual ability score.

### Data Acquisition and Analyses

A Tobii X2–60 Hz eye tracker recorded each child’s pupil diameter in response to the affective images. Pictures were presented on a laptop screen that was 55.9 cm with a screen resolution of 1680 × 1080 pixels, positioned approximately 60 cm from the child’s eyes, in a dim room. The data was cleaned and analyzed using Matlab (MathWorks, version 8.5) based on the methods described in Burley et al. (2017).

We were unable to obtain pupillometry data for six children (due to difficulties sitting still, a lack of engagement, etc). Also, children with less than 50% valid data across all trials during image presentation were excluded leading to the removal of nine participants. This left a sample of 116 participants (33 girls).

The split-half internal reliability was high for mean pupil diameter during image presentation (*r* = .99, using a Spearman-Brown correction).

Pupil diameter was recorded in millimetres. To identify changes in pupil diameter specific to the affective component of the negative and positive images respectively, we calculated the difference in pupil diameter to each affective category compared to neutral images. This was calculated from 1000 to 2000 ms following image presentation as pupil response over this later period is considered to primarily reflect sympathetic activation (Bradley et al. 2017). We ran correlations assessing the relationships between negative–neutral and positive-neutral pupil diameter and SDQ scores. We controlled for each individual’s overall pupil diameter within these correlation analyses, defined as pupil size over the period 200 ms prior to image presentation (Burley et al. 2018), as well as participant age. We note that there was no difference in pupil diameter in response to negative or positive images (minus pupil diameter to neutral images) across participant gender (*p*s > .58) and so we did not include this variable in analyses.

## Results

**Fig. 1:**
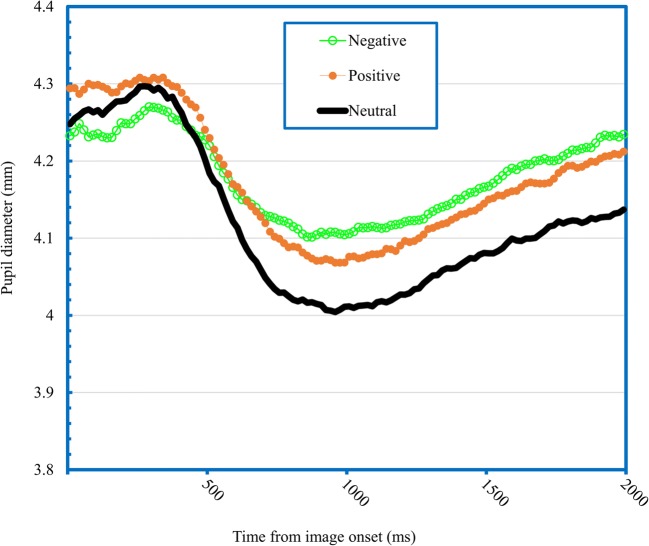
Pupil diameter in response to negative, positive and neutral images across the sample of young children (*n* = 116)

Figure 1 shows the change in pupil diameter in response to the onset of the images, revealing a typical pattern within the sample of increased pupil diameter to negative and positive images compared to neutral images. Mean pupil diameter was calculated in response to negative, positive and neutral images from 1000 to 2000 ms post-image onset and a repeated measures ANOVA revealed a main effect of emotion, *F*(2, 230) = 24.98, *p* < .001, *η*^*2*^ = .18, 90% CI [.10, .24]. Both negative (M = 4.16, SD = 0.56) and positive images (M = 4.14, SD = 0.55) led to greater pupil diameter than neutral images (M = 4.07, SD = 0.53) respectively (*p*s < .001), with no difference between negative and positive images (*p* = .16).

Child estimated intelligence, as measured by the Lucid Ability assessment, was not related to pupil diameter in response to negative or positive images (minus pupil diameter to neutral images) (*p*s > .26) and so was not considered further in subsequent analyses.

### Emotionally-Modulated Pupil Response and SDQ Scores

Table 1 reports the SDQ scores of the participants for whom we obtained pupil data. Table 2 describes the relationships between the SDQ subscales. Scores on the Conduct problems, Hyperactivity and Peer problems subscales were significantly positively related, with these subscales showing negative associations with the Prosocial subscale. The SDQ Emotional problems subscale showed a positive relationship with the Conduct subscale, with no relationships emerging with scores on the other dimensions.

**Table 1:** Descriptive information for the Strengths and Difficulties Questionnaire (SDQ) scores of the participants for whom we obtained pupil data (*n* = 116)

Variable	M (SD)	High-risk ^†^ (%)
SDQ subscales		
Emotional problems	3.22 (2.60)	31.90
Conduct problems	3.39 (2.59)	44.80
Hyperactivity	7.63 (2.31)	59.50
Peer problems	3.31 (2.32)	35.30
Prosocial	4.51 (2.89)	53.40
Total difficulties	17.55 (6.33)	65.50
Disruptive behaviour	4.96 (1.93)	

^†^High risk was defined as scores within the ‘High’ or ‘Very High’ categorisation and ‘Low’ and ‘Very Low’ for the SDQ Prosocial subscales according to the SDQ scoring guidelines retrieved from http://www.sdqinfo.org/py/sdqinfo/b3.py?language=Englishqz(UK)

*Note.* Composite Disruptive behaviour scores were calculated from the mean score across the Conduct, Hyperactivity, Peer problems and reversed Prosocial behaviour subscales

**Table 2:** Pearson’s correlations between the Strengths and Difficulties Questionnaire (SDQ) subscales scores for whom we obtained pupil data (*n* = 116)

Subscales	1	2	3	4
1. Emotional problems				
2. Conduct problems	.20* [.02, .37]			
3. Hyperactivity	−.04 [−.22, .14]	.44*** [.28, .58]		
4. Peer problems	.06 [−.12, .24]	.40*** [.24, .54]	.27* [.09, .43]	
5. Prosocial	−.01 [−.19, .17]	−.59***[−.70, −.46]	−.40** [−.54, −.24]	−.52** [−.64, −.37]

* *p* < .05, ** *p* < .01, *** *p* < .001

[95% CI], confidence intervals are presented for correlations

We assessed the relationship between SDQ scores and both negative-neutral and positive-neutral pupil diameter over 1000–2000 ms post-image onset, while controlling for each participant’s overall pupil size and age. As can be seen in Table 3, negative-neutral pupil diameter was inversely related to Emotional problems, Conduct problems, Hyperactivity and Peer problems, and positively related to Prosocial behaviour. Not surprisingly, SDQ Total difficulties scores were inversely associated with negative-neutral pupil diameter. A multiple linear regression revealed that only Emotional problems uniquely and negatively predicted negative-neutral pupil diameter (*t*(110) = −2.41, *p* = .02, *β* = −.23, 95% CI [−.41, −.04]), with none of the remaining SDQ subscales being predictive (*p*s > .25). Given the overlap between the Conduct problems, Hyperactivity, Peer problems and Prosocial behaviour subscales, we created a composite score consisting of the mean scores across these subscales that we termed ‘Disruptive behaviour’. Negative-neutral pupil diameter was inversely related to Disruptive behaviour (see Table 3). When we entered this score alongside Emotional problems as predictors into a multiple linear regression (the *α-*level was Bonferroni-adjusted for these additional analyses based on two predictor variables) we found that both Emotional problems (*t*(112) = −2.74, *p* = .01, *β* = −.24, 95% CI [−.43, −.07]) and Disruptive behaviour (*t(*112) = −2.38, *p* = .02, *β* = −.21, 95% CI [−.39, −.04]) uniquely negatively predicted negative-neutral pupil diameter.

**Table 3:** The relationship between Strengths and Difficulties Questionnaire (SDQ) scores and negative/positive-neutral pupil diameter (*n* = 116), controlling for overall pupil size and participant age

Variable	*df*	Negative-neutral pupil diameter	Positive-neutral pupil diameter
		*r* [95% CI]	*r* [95% CI]
SDQ subscales			
Emotional problems	112	−.23* [−.40, −.05]	−.14 [−.31, .04]
Conduct problems	112	−.23* [−.40, −.05]	−.04 [−.22, .14]
Hyperactivity	112	−.19* [−.36, .01]	−.10 [−.28, .08]
Peer problems	112	−.24** [−.36, −.06]	−.05 [−.23, .13]
Prosocial	112	.23* [.05, .40]	.07 [−.11, .25]
Total difficulties	112	−.34*** [−.49, −.17]	−.13 [−.31, .05]
Disruptive behaviour	112	−.29** [−.45, −.11]	−.09 [−.27, .10]

* *p* < .05, ** *p* < .01, *** *p* < .001

[95% CI], confidence intervals are presented for correlations

*Note.* Composite Disruptive behaviour scores were calculated from the mean score across the Conduct, Hyperactivity, Peer problems and reversed Prosocial behaviour subscales

As can be seen in Table 3, none of the SDQ subscales showed a relationship to positive-neutral pupil diameter.

### General Discussion

The aim of the current study was to examine whether abnormal autonomic arousal to affective stimuli measured using pupillometry was related to severity of conduct and emotional problems in a high-risk sample of young children showing early signs of psychopathological problems, and whether impaired reactivity was specific to negative stimuli. Firstly, we observed increased pupil diameter across our sample in response to negative and positive images compared to neutral images, consistent with ‘normal’ pupil response patterns collected previously within community samples (Bradley et al. 2008; Snowden et al. 2016). More importantly, reduced pupil dilation to negative images was associated with increased conduct symptoms. These findings are consistent with previous research that has indicated that low autonomic arousal to aversive cues is associated with risk of aggression or antisociality later in life (Baker et al. 2013; Gao et al. 2009, 2010) and findings in antisocial adults using pupillometry (Burley et al. 2018). The autonomic hypo-reactivity observed among children with an early disruptive profile may drive their antisocial development, as they are insensitive to negative cues and fail to recognise or respond to the negative consequences associated with behaviour (van Goozen 2015). This conceivably could explain the child’s preparedness to engage in and persist with antisocial behaviours, as well as their failure to withdraw from threatening or aversive situations. The current study is the first to demonstrate reduced pupil reactivity to negative stimuli as a biomarker of conduct problem severity in children.

Interestingly, the pattern of blunted pupil activity to negative stimuli was not limited to the dimension of conduct symptoms alone; it was also associated with elevated emotional difficulties, hyperactivity, peer problems, and diminished prosocial behaviour. In fact, no subscale, aside from emotional problems, uniquely predicted blunted pupil responsivity to the negative threat images alone. This may be due to the high degree of overlap between conduct, hyperactivity, peer and prosocial problems, which is unsurprising given the co-occurrence of externalising difficulties (Lahey et al. 2008; Patalay et al. 2015). When we created a single disruptive behaviour scale (comprising conduct, hyperactivity, peer and prosocial problems) this dimension indeed predicted blunted pupil reactivity to negative threat stimuli alongside the Emotional problems subscale. This suggests that reduced pupil responses to negative threat stimuli is an autonomic biomarker within young children for an early profile of disruptive behaviour, including conduct problems, hyperactivity and interpersonal difficulties, as well as reduced prosocial behaviours that has been linked to early emerging psychopathic traits (Dadds et al. 2005).

The current study also found that severity of emotional problems in young children uniquely predicted the pattern of diminished pupil reactivity to negative images. This finding is consistent with previous pupillometry research that reports that depression severity (within adolescents and adults) was related to diminished pupil dilation to negatively-laden words (Siegle et al. 2011; Silk et al. 2007). Siegle et al. (2011) also reported that increased pupillary responses were correlated with activity in the dorsolateral prefrontal region, a region associated with executive control and emotion regulation, in individuals with high levels of depressive symptoms (as well as control participants). It was therefore argued that individuals with elevated internalising symptoms may be characterised by disrupted executive control mechanisms responsible for emotional regulation reflected in reduced pupil reactivity. Therefore, diminished pupillary dilation to negative threat stimuli, as observed in the present study, can act as an autonomic biomarker of emotional difficulties in childhood, that may reflect underlying emotional dysregulation.

This finding suggests that low ANS may be a risk factor for early maladjustment including internalising difficulties (Chen et al. 2015), which is consistent with recent conceptualisations that psychopathology within children (likewise to adult mental health) is characterised by a general psychopathology risk factor that bridges externalising and internalising difficulties (Patalay et al. 2015). However, there are alternative studies that have reported that internalising difficulties in the context of additional childhood conduct problems are associated with heightened emotionality and hyper-arousal (Fanti and Kimonis 2017; Fanti et al. 2018). However, these studies used participants from community samples rather than high-risk samples such as the current study. The children in the current study had complex backgrounds and were already experiencing substantial early difficulties across a range of emotional, behavioural and interpersonal dimensions - to the point where teachers working with them made a referral to the NDAU seeking help – and therefore represent a pre-diagnostic developmental sample. The complex and overlapping nature of the difficulties present in our high-risk sample seem likely to differ from those present in community samples where functioning is higher and the nature of emotional problems (as well as wider difficulties) are less severe, which may account for differential patterns of autonomic arousal in relation to internalising difficulties. A further reason for the discrepancy in findings for the emotional problems dimension is that previous studies tend to create subgroups high in conduct/callous symptoms with high or low internalising difficulties. Treating these difficulties as orthogonal is surprising and fails to recognise the comorbidity of externalising and internalising difficulties (Nock et al. 2007) in contrast to the current dimensional approach. Further, a groups-based approach loses the heterogeneity that exists within specific constructs which may mask independent dimensional relationships, which is particularly relevant given the distinct and varying patterns of physiological reactivity observed within conduct and anxiety spectrums (Fanti 2016; Lang et al. 2016).

Despite individuals with conduct problems showing both aberrant threat and reward processing (Byrd et al. 2014), our findings indicate that early conduct problems (as well as wider behavioural, emotional and interpersonal difficulties) are selectively related to abnormal pupil responses to negative images with no association emerging for pupil response to positive images (matched for subjective arousal ratings), similar to findings in adult psychopathy (Burley et al. 2018). Given that arousal is an indicator of the sensitivity of an organism to emotional cues within the RDoC framework, this suggests that early conduct problems are related to a specific insensitivity to negative cues, alongside typical autonomic reactivity to positive cues. This provides evidence that the reward-oriented behaviours associated with antisociality do not occur due to an abnormal autonomic response to positive cues (Fairchild et al. 2009), but rather may reflect a failure to respond appropriately to negative cues (i.e., fearlessness) consistent with a weakened defensive motivation system. Conversely, Burley et al. (2018) reported that psychopathy traits within adults were associated with increased pupil diameter in response to happy faces, indicating stimulus-specific effects that need further investigation.

### Implications

The current study highlights reduced pupil reactivity to negative cues as a biomarker linked to conduct problems and wider disruptive behaviour difficulties, as well as emotional problems within childhood. This is important as longitudinal studies indicate that infants who show early blunted autonomic responsivity are at greater risk of engaging in future antisocial and aggressive behaviours (Baker et al. 2013; Gao et al. 2009, 2010; van Goozen 2015). Early neurobiological biomarkers, such as blunted pupil responses to negative cues, may help to identify children early who are at risk of more severe conduct or emotional difficulties later. Opportunities for early intervention are promising because of the greater plasticity of the brain early in life (Cioni et al. 2016; Ismail et al. 2017), which may offer immediate benefits as well as potential disruption to a pathway to later psychopathology and/or antisociality. In addition, our results highlight the value of a dimensional over traditional case-control approach to neurodevelopmental difficulties (i.e., extreme variants of normal personality variables) and in relation to autonomic reactivity. It is important that future research adopts and extends the dimensional approach when using autonomic measures, such as pupil responsivity, as clustering variables consistent with the RDoC framework to allow for greater understanding of the variables that contribute to abnormal sympathetic autonomic arousal.

### Limitations

This study has several limitations. The children were seen at a single time-point, limiting the current study’s ability to assess the predictive value of pupil reactivity to affective cues for conduct and emotional problems across development. The sample was comprised of young children referred by their schools for emotional and behavioural difficulties, and because of their young age none of the children had yet received a formal diagnosis. However, given the severity of the problems reported, it seems likely that many will receive a diagnosis later in life.

## Conclusion

The current findings indicate that reduced pupil responsivity to negative stimuli is a biomarker of early conduct, affective and interpersonal difficulties, as well as reduced prosocial behaviours in young children, indexing a possible future antisocial profile. No similar autonomic insensitivity was observed in response to positive stimuli. The current research highlights the value of the current pupillometry paradigm as a fast, time-sensitive, and non-intrusive measure to identify young children experiencing early difficulties, which can provide opportunities for intervention to disrupt pathways to later psychopathology.

## Electronic supplementary material

ESM 1 (SAV 24 kb)
